# Effects of different light spectra on locomotion, anxiety, and heart rate in spontaneously hypertensive rats and Wistar–Kyoto rats

**DOI:** 10.1016/j.ibneur.2025.11.008

**Published:** 2025-11-19

**Authors:** Teng-Ching Yin, Meng-Li Tsai, Shih-Han Liao, Wen-Yi Wu

**Affiliations:** a Department of Biomechatronic Engineering, National Ilan University, Yilan County 26047, Taiwan; b School of Medicine, College of Medicine, Fu Jen Catholic University, New Taipei City 242062, Taiwan

**Keywords:** Attention-deficit/hyperactivity disorder, Light therapy, Heart rate variability

## Abstract

Light exposure plays a critical role in regulating physiological and behavioral processes. This study aimed to explore how different light spectra influence locomotor activity, anxiety-like behavior, and heart rate variability in rodents. We used spontaneously hypertensive rats (SHRs), which exhibit distinct behavioral traits compared to normotensive Wistar–Kyoto (WKY) rats, as a model to examine strain-related differences in response to light conditions. Given known sex differences in behavioral phenotypes and light sensitivity, and the higher prevalence of certain behavioral traits in males, this study focused on male rats. Animals were exposed to white, blue, green, or red light-emitting diodes (LEDs), and their behavioral and physiological responses were assessed through locomotion tracking and electrocardiography. The results showed that both light spectrum and intensity significantly influenced activity levels, anxiety-like behavior, and heart rate in a strain-dependent manner. These findings provide insights into how spectral qualities of light affect behavior and physiology, and suggest potential considerations for light environment design in future behavioral and translational studies.

**Significant statements:**

Blue light consistently suppressed motor activity and increased anxiety-like behavior in SHRs, who also showed greater heart rate elevations and heightened autonomic sensitivity to light compared to WKY rats.

## 1 Introduction

Light plays a crucial role in regulating circadian rhythms, behavior, and physiological processes in both humans and animals. Advances in artificial lighting have extended activity into nighttime hours and prompted investigations into how light exposure influences mood, stress, and behavior. Different light spectra and intensities are known to differentially affect neuroendocrine function, emotional regulation, and cognitive performance, with implications for both health and disease (Bedrosian and Nelson, 2013, LeGates et al., 2014). Irregular light exposure can disrupt circadian rhythms, affect hormonal balance, and increase the risk of stress-related disorders (Bedrosian et al., 2016, Fonken et al., 2010).

Rodent models provide a useful framework for studying the behavioral and physiological effects of environmental lighting. Among them, spontaneously hypertensive rats (SHRs) exhibit characteristic hyperactivity, impulsivity, and altered stress reactivity (Adriani et al., 2003), distinguishing them from normotensive control strains such as Wistar–Kyoto (WKY) rats (Grundt et al., 2009). These behavioral differences have made SHRs a valuable model for exploring neurobehavioral regulation and strain-dependent responses to environmental stimuli. Notably, male rats have been widely used due to consistent expression of these behavioral traits and sex-related differences in light sensitivity and stress response (Krizo and Mintz, 2014).

As nocturnal animals, rats possess a visual system primarily composed of rod cells, with a small proportion of cone cells responsive to specific light wavelengths. In albino rats, approximately 7 % of all cones are short-wavelength (S) cones, which are sensitive to ultraviolet (UV) light (Szél and Röhlich, 1992). However, the majority are medium-to-long-wavelength cones, which are sensitive to green light (Jacobs et al., 2001, Jacobs et al., 1991). Environmental brightness and light color significantly influence rat behavior and circadian rhythms, with different wavelengths exerting different effects (Bedrosian et al., 2013). Heart rate variability (HRV), a key indicator of autonomic nervous system activity, reflects the balance between sympathetic and parasympathetic regulation. HRV is typically assessed through electrocardiography (ECG). It is a noninvasive and reliable measure of physiological stress response (Berntson et al., 1997, Saul et al., 1989). Changes in HRV are widely assessed in studies investigating the association between the autonomic nervous system function and cardiovascular health. Thus, HRV a valuable metric in psychophysiological research (Chapleau and Sabharwal, 2011, Havlicekova et al., 2009, Karmakar et al., 2012, Marek, 1996).

In this study, we investigated how different light spectra of light-emitting diodes (LEDs)—white, blue, green, and red—affect locomotor activity, anxiety-like behavior, and HRV in male SHRs and WKY rats. By comparing the behavioral and physiological responses of these two strains under varying light conditions, we aimed to understand how environmental lighting influences emotional and physiological regulation. We hypothesized that light spectrum and intensity would differentially affect these outcomes in a strain-dependent manner, with short-wavelength light (e.g., blue) expected to produce more pronounced behavioral and autonomic effects.

## 2 Methods

### 2.1 Animals

For this study, we used 7-week-old male SHRs (n = 23) and WKY rats (n = 23). The rats were purchased from BioLASCO Taiwan. The animals were housed under a 12-h light–dark cycle (lights were on at 6 AM) and maintained at a controlled ambient temperature. All rats had free access to food and water and were housed in the institutional animal facility. All experiments were conducted during the dark phase. Animal care and handling as well as all experimental procedures adhered to the Codes for Experimental Use of Animals outlined by the Council of Agriculture, Taiwan, on the basis of Animal Protection Law, Taiwan. The study protocol was approved by the Institutional Animal Care and Use Committee of National Ilan University (Approval No. 103–37).

### 2.2 Surgery

Rats were anesthetized with pentobarbital sodium (0.1 mL/100 g body weight; 60 mg/mL), and the fur over the dorsal neck and axillary regions was shaved. A 10-mm incision was made at the dorsal neck and three additional 2-mm incisions were created at the left and right axillary regions and the central area between them. The connective tissue between the skin and underlying musculature was gently separated to create a subcutaneous pocket for implantation of the ECG device, following the method established by Tsai et al. (2007). The device, consisting of three electrodes individually welded to signal wires, was then inserted: two electrodes recorded the differential potential, while the third served as the ground. The insulated stainless steel wires (Bare: 25.4 μm, Coated: 139.7 μm) were routed through the smaller incisions and secured with sutures. All incisions were subsequently closed and treated with neomycin ointment to prevent infection, and the animals were placed on a heating blanket until they regained consciousness.

### 2.3 Procedure

Following surgery, 1 day recovery period was provided. Prior to testing, the rats were given and additional 2 days to acclimate to the experimental arena. The light conditions were systematically varied in the following sequence: dark, white, blue, green, and red. Although experiments commenced 1 day after the operation and continued for 2 weeks, ECG recordings were not collected until postoperative day 4. The rats were merely placed in the experimental cages on days 2 and 3 to adapt to the environment. Notably, the surgery involved only a minor procedure—suturing a lightweight electrode beneath the skin—unlike more invasive operations such as craniotomy. In addition, the postoperative rest period was sufficient for complete metabolism of the anesthetic agent. ECG recordings were obtained throughout the experimental period. HRV was analyzed using a low-frequency (LF) band (0.06–0.6 Hz) and a high-frequency (HF) band (0.6–2.4 Hz), in accordance with established guidelines from previous HRV studies in SHRs (Kuo TB et al., 2004).

The experimental protocol consisted of two groups: one exposed to increasing illuminance (SHR, n = 12 rats; WKY, n = 12 rats) and the other to decreasing illuminance (SHR, n = 11 rats; WKY, n = 11 rats). Each rat was first given a 10-minute acclimation period in the open field. After acclimation, lighting conditions were initiated, and each light intensity lasted for 10 min. During this period, both locomotion tracking and ECG recording systems were simultaneously activated. At the end of each session, the equipment was turned off, and the amount of defecation was recorded. The arena was then cleaned using an alcohol-water solution. After a 5-minute interval, the rat was placed in the open field again for exposure to the next light intensity. No white noise was provided; only ambient noise was present. The room temperature was maintained at 24°C.

### 2.4 Light exposure parameters

LEDs offer several advantages over traditional lighting systems—for example, a lower calorific value, which indicates the thermal energy emitted by a lamp. Additionally, LEDs produce a pure color spectrum with a narrow spectral bandwidth (range: 5–50 nm) (Geng X et al., 2017). In this study, the LED lamp holder was mounted on an aluminum shelf measuring 66 cm × 80 cm × 102 cm. The holder’s height was adjustable, ensuring uniform illuminance across color temperatures. Illuminance levels were categorized as low (21–31 lux) and high (161–171 lux).

The uniformity of light distribution within a given space can be quantified by assessing the uniformity of illuminance. The ratio of minimum illuminance to average illuminance serves as an indicator of spatial uniformity. This ratio ranges from 0 to 1, with a value of 0 indicating minimal uniformity and a value of 1 indicating complete uniformity. To measure this parameter, the arena floor (50 cm × 50 cm) was divided into 25 squares. The illuminance of each square was measured (Table 1).

$$\mathrm{Uniformity}=\frac{\mathrm{minimum illuminance}}{\mathrm{average illuminance}}$$

**Table 1:** LED specifications and uniformity of light distribution.

	White	Blue	Green	Red
Brand	TOA lighting	VITALUX	VITALUX	VITALUX
Model	LTU004–9AAD-E	custumized	custumized	custumized
Type	LED T9 lamp	LED T8 lamp	LED T8 lamp	LED T8 lamp
Size (Feet)	2	2	2	2
Power (W)	9	16–17	16–17	12–15
Wavelength (nm)	-	440–455	520–535	655–665
Uniformity of low lux	0.86	0.85	0.78	0.72
Uniformity of high lux	0.86	0.9	0.82	0.8

### 2.5 Open field test and locomotion

A square, transparent, acrylic arena measuring 50 cm × 50 cm × 100 cm was divided into identical virtual grids (7 × 7), establishing 40 peripheral zones and 9 central zones. The central zone accounted for approximately 16 % of the total floor area of the arena. A Kinect sensor was positioned above the arena, with its camera directed toward the floor. The arena was enclosed within a structure made of gypsum and wood (96 cm × 100 cm × 137 cm) to minimize ambient noise. This enclosure effectively reduced environmental noise levels from 36 to 17 dB. During the experiment, the rats were individually placed in the central zone of the arena.

In the experimental arena, rat locomotion was primarily categorized into two distinct behaviors: walking and exploration. Walking was defined as any movement exceeding 5 cm within a 2-s interval. Movements failing to meet this criterion were classified as exploration, which included behaviors such as rearing, grooming, and paw licking, which were monitored through color video recording. An automated, three-dimensional locomotion tracking and pose reconstruction system was used for measuring locomotion, as described previously (Ou-Yang TH et al., 2011). This system enables the tracking of rat movement and the reconstruction of virtual images across three orthogonal planes (XY, YZ, and XZ). The system employs an infrared camera and remains unaffected by variations in ambient lighting, fur color, or complex backgrounds, thereby ensuring consistent experimental conditions throughout the study.

### 2.6 Statistical analysis

**Fig. 1:**
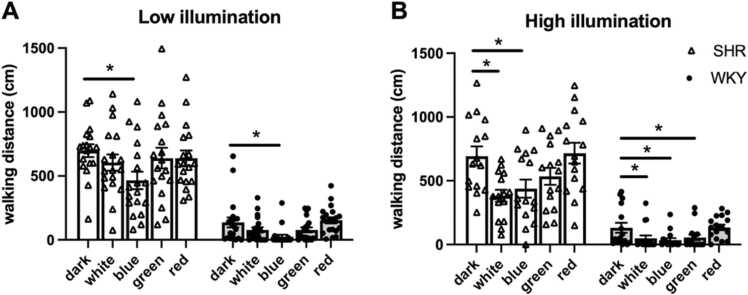
Motor activity in SHRs (black bar) and WKY rats (white bar) under low (A) and high (B) illumination across different colored light conditions. (A) Under low illumination, the walking distance in both SHRs and WKY rats significantly decreased under blue light compared with the findings obtained under the dark condition (SHR: *n* = 18; WKY: *n* = 20). (B) Under high illumination, the walking distance in both SHRs and WKY rats significantly decreased under blue and white light compared with the findings obtained under the dark condition (SHR: *n* = 15; WKY: *n* = 16). Data are presented in terms of mean ± SEM values. *p < .05, compared with the dark condition.

Data were analyzed using a two-way repeated-measures analysis of variance (ANOVA), with strain (SHR vs. WKY) and illumination intensity (low vs. high) used as between-subject factors and light color (five levels) as a within-subject factor. When significant main effects or interactions were identified, post hoc comparisons were performed using Bonferroni correction to adjust for multiple comparisons. Data are presented in terms of mean ± standard error of the mean (SEM) values. For Fig. 1, statistical comparisons of locomotor activity under different colored light conditions were conducted using paired *t*-tests, with the dark condition serving as the baseline for each animal. Separate analyses were performed for low and high illumination settings within each strain. A significance level of *p* < .05 was considered statistically significant.

## 3 Results

### 3.1 Reduced motor activity in rats exposed to blue light

Motor activity was evaluated by measuring total movement distance, rearing frequency, standing duration, and horizontal movements over a 10-min period. Statistical analyses were performed to evaluate the effects of various light colors on SHRs and WKY rats under both high- and low-illumination conditions. Under low illumination, the average activity levels across all light conditions were lower than those observed under the dark condition, with the most pronounced reduction occurring under blue light. This effect was statistically significant, as determined using a two-way repeated-measures ANOVA (F [4, 33] =9.002, *p* < .001 partial η² =.522; Fig. 1A). Similarly, under high illumination, a significant effect of light color on activity levels was observed (F[4, 26] = 6.613, p < .001, partial η² =.504; Fig. 1B). Post hoc comparisons (Bonferroni-corrected) revealed that average activity levels in both rat strains were significantly lower under blue and white light compared to the dark condition, whereas no significant differences were found for green and red light.

Across all light colors, SHRs exhibited significantly higher activity levels than did WKY rats under both low (F [1, 36] = 97.538, *p* < .001, partial η² =.73) and high illumination (F [1, 29] = 82.952, *p* < .001, partial η² =.838; Fig. 1). Among the different light conditions, blue light resulted in the lowest activity levels for both strains, with a significant main effect of color under low (F [4, 144] = 7.757, *p* < .001, partial η² =.177) and high illumination (F [4, 116] = 12.171, *p* < .001, partial η² =.296; Fig. 1). Although overall activity levels differed significantly between strains and across light colors, the pattern of responses to the different light conditions was largely similar in both strains. This was supported by a nonsignificant interaction between strain and light color under low illumination (F[4, 144] = 1.166, p = .328, partial η² =.031), and a relatively weak interaction under high illumination (F[4, 144] = 3.462, p = .010, partial η² =.107; Fig. 1). Furthermore, walking distances did not significantly differ between the low- and high-illumination conditions across any light color in either strain. This was confirmed by two-way repeated-measures ANOVA, which revealed no significant effect of illumination in SHRs (F[4, 28] = 2.530, p = .063, partial η² =.265) or WKY rats (F[4, 31] = 0.433, p = .783, partial η² =.053; Figure S1).

### 3.2 Differential anxiety responses in SHRs and WKY rats under blue light condition

**Fig. 2:**
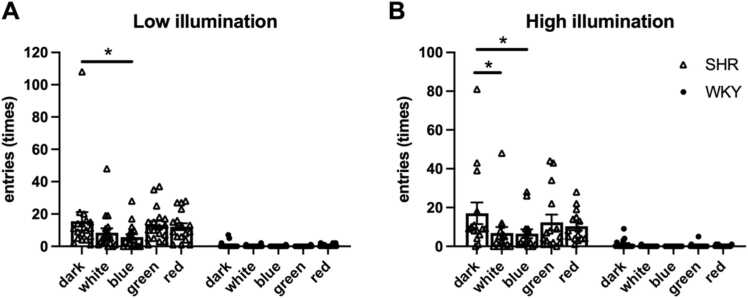
Frequency of entries into the central zone of the open field in SHRs (black bar) and WKY rats (white bar) under low-illumination conditions (A) and high-illumination conditions (B) across all colored light conditions. (A) Under low illumination, SHRs (*n* = 18) exhibited a significantly higher number of entries into the central zone than WKY rats (*n* = 20). (B) Under high illumination, SHRs (*n* = 15) exhibited significantly higher entry frequencies than WKY rats (*n* = 16). Data are presented in terms of mean ± SEM values. *p < .05, compared with the dark condition.

Across all light conditions and illumination intensities, SHRs exhibited significantly higher frequencies of entries into and exits from the central field than did WKY rats. This effect was observed under both low (F [1,36] = 34.875, *p* < .001, partial η² =.492) and high illumination (F [1,29] = 14.379, *p* < .001, partial η² =.331; Fig. 2). This increased locomotor activity in SHRs was the most pronounced under the dark condition and then under green, red, white, and blue light. By contrast, WKY rats consistently exhibited low activity levels across all colored light conditions. A significant interaction between light color and rat strain was observed under both low (F [4,33] = 11.193, *p* < .001, partial η² = 0.576) and high illumination (F [4,26] = 8.251, *p* < .001, partial η² =.639). These interactions, characterized by large effect sizes, indicate distinct strain-specific responses to various colored light stimuli. Notably, SHRs demonstrated significant reductions in activity on exposure to blue light under low-illumination conditions and on exposure to both blue and white light under high-illumination conditions compared with the findings under the dark condition. Conversely, WKY rats exhibited no significant differences in activity levels across light conditions (Fig. 2). These findings suggest that blue light differentially influences anxiety in SHRs and WKY rats. Moreover, light intensity exerted no significant effect on either strain under any light condition (SHR: F [1,32] = 0.015, *p* = .903, partial η² <.001; WKY: F [1,34] = 0.437, *p* = .513, partial η² = 0.013; Figure S2). This finding indicates that although SHRs and WKY rats exhibit distinct locomotor responses to colored light stimuli, their anxiety-like behaviors remain relatively stable across illumination intensities.

### 3.3 Effects of colored light on heart rate in SHRs and WKY rats

**Fig. 3:**
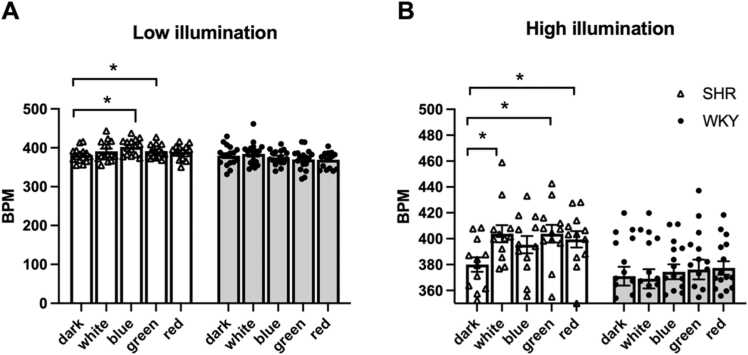
Differential responsiveness of SHRs and WKY rats to colored light stimulation under low (A) and high (B) illumination. (A) Under low illumination, SHRs (*n* = 15) exhibited a significant response to blue and green light, whereas WKY rats (*n* = 17) did not demonstrate significant differences in response across any of the colored light conditions. (B) Under high illumination, SHRs (*n* = 12) exhibited significant responses to white, green, and red light. By contrast, WKY rats (*n* = 16) exhibited no significant differences in response among the colored light conditions. Data are presented in terms of mean ± SEM values. *p < .05, compared with the dark condition.

Under low-illumination conditions, SHRs exhibited significantly higher heart rates than WKY rats across all light colors (F [1, 30] = 7.063, *p* = .012, partial η² =.191). A significant interaction between rat strain and colored light was observed (F [4,27] = 2.930, *p* = .039, partial η² =.303). Post hoc analysis with Bonferroni correction revealed that exposure to blue and green light led to a significant increase in heart rate in SHRs, indicating that specific wavelengths may exert strain-dependent cardiovascular effects (Fig. 3A).

Under high-illumination conditions, SHRs exhibited significantly higher heart rates than WKY rats across all light colors (F [1, 26] = 9.751, *p* = .004, partial η² =.273). A significant main effect of color was observed on heart rate (F [4, 23] = 2.969, *p* = .041, partial η² =.341). Although the interaction between strain and colored light was nonsignificant (F [4, 23] = 2.568, *p* = .065, partial η² = 0.309), the data suggest that WKY rats exhibited minimal HRV across colors, whereas SHRs appeared to be more responsive to color changes (Fig. 3B). Despite the significant effect of colored light on heart rate, illumination intensity itself did not exert a significant effect on either strain (SHR: F [1, 25] =.854, *p* = .364, partial η² =.033); WKY: (F [1, 31] =.097, *p* = .757, partial η² =.003; Figure S3).

### 3.4 Enhanced parasympathetic response to high-intensity white light in SHRs

**Fig. 4:**
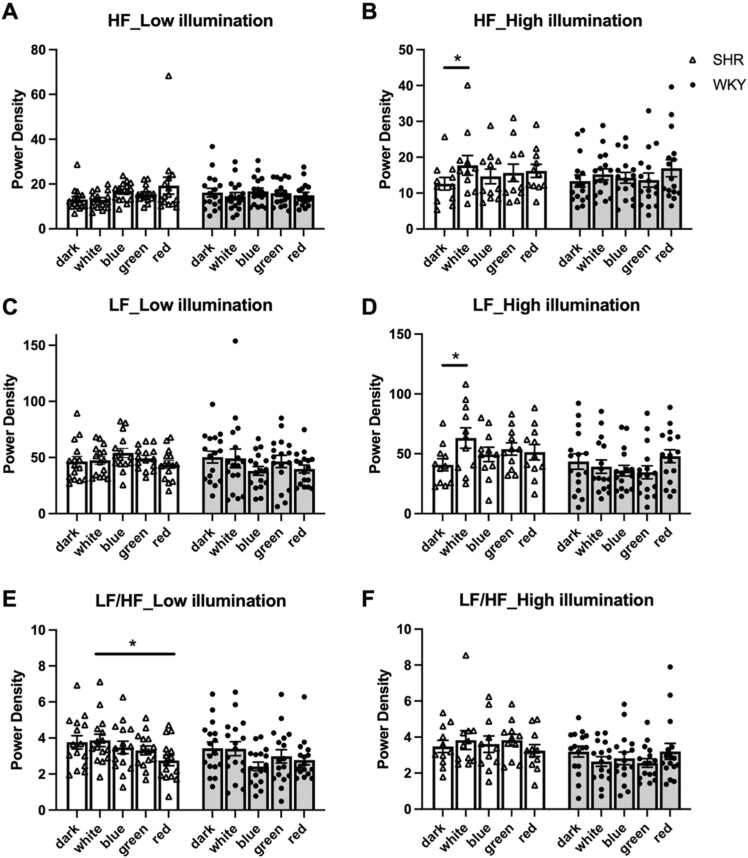
Effects of light color and intensity on HRV parameters in SHRs and WKY rats. (A and B) Low-frequency (LF) power density under low- and high-illumination conditions. (C and D) High-frequency (HF) power density under low- and high-illumination conditions. (E and F) LF/HF ratio under low- and high-illumination conditions. Black bars represent SHRs, and white bars represent WKY rats. Low illumination: SHR, *n* = 15; WKY, *n* = 17. High illumination: SHR, *n* = 11; WKY, *n* = 16. Data are presented in terms of mean ± SEM values. *p < .05.

To investigate the effect of colored light on autonomic nervous system regulation, HRV was measured across different light conditions, focusing on three key parameters: LF, HF, and the LF/HF ratio. HRV was quantified using power spectral density (ms²/Hz), which provides a frequency-domain description of the variance in R–R intervals. The integrated LF and HF components (ms²) reflect sympathetic and parasympathetic contributions, respectively, and thus the observed spectral changes highlight the impact of light on autonomic regulation. Under low-intensity light conditions (Fig. 4A), no significant interaction was observed between light color and strain (*p* = .272). However, light color exhibited a moderate effect size (partial η² = 0.168). Under high-intensity light conditions (Fig. 4B), a significant main effect of light color was observed on LF (*p* = .028, partial η² =.102), along with a significant interaction between light color and strain (*p* = .028, partial η² =.102). Notably, SHRs exhibited substantially higher LF values under white light (63.1 ± 8.2) than under the dark condition (40.8 ± 6.1), suggesting an enhanced sympathetic activation in response to bright white light. By contrast, WKY rats exhibited relatively stable LF values across all light conditions, with slightly elevated values under the dark (43.5 ± 5.8) and red light (47.9 ± 6.3) conditions. These findings indicate that SHRs are more sensitive than WKY rats to light-induced changes in sympathetic nervous system activity, particularly under high-intensity white light.

Under low-intensity light conditions (Fig. 4C), the effect of light color on HF approached significance (*p* = .067, partial η² =.270). Under high-intensity light conditions (Fig. 4D), light color had a significant effect on HF (*p* = .021, partial η² =.395), with white light eliciting significantly higher HF responses than did the dark condition (*p* = .012). Specifically, in SHRs, the HF values increased from 12.5 ± 2.1 under the dark condition to 17.7 ± 2.8 under white light, indicating a marked increase in parasympathetic activity. WKY rats exhibited a similar, but less pronounced, trend, with HF values increasing from 13.3 ± 1.9 under the dark condition to 15.2 ± 1.7 under white light. The more pronounced response in SHRs suggests that high-intensity white light exerts a strong modulatory effect on parasympathetic tone in hypertensive rats. This differential response between strains may have important implications for understanding autonomic regulation in hypertensive conditions and may inform light therapies for managing autonomic dysfunction associated with hypertension.

Under low-intensity light conditions (Fig. 4E), light color exerted a significant effect on the LF/HF ratio (*p* = .002, partial η² =.133), with white light eliciting significantly higher ratios than did red light (*p* = .015). Under high-intensity light conditions (Fig. 4F), no significant effect of light color was observed on the LF/HF ratio (*p* = .990, partial η² =.003). However, the difference between SHRs and WKY rats approached significance (*p* = .059, partial η² =.135). Notably, SHRs exhibited consistently higher LF/HF ratios across most light conditions than did WKY rats, with the most pronounced differences observed under white light (3.8 ± 0.3 vs. 3.4 ± 0.4) and green light (3.3 ± 0.3 vs. 2.9 ± 0.3) under low-illumination conditions. These results indicate a predisposition toward sympathetic dominance in SHRs, particularly under specific light conditions. The near-significant between-strain difference under high-illumination conditions further implies that genetic predisposition to hypertension influences autonomic responses to environmental stimuli, with SHRs consistently exhibiting higher sympathetic predominance than do their normotensive counterparts. These findings highlight the potential role of light exposure in differentially modulating autonomic balance in hypertensive and normotensive subjects.

## 4 Discussion

This study provides evidence that light spectrum and intensity influence both behavioral and autonomic outcomes in rodents, with SHRs showing greater sensitivity than WKY rats. Such strain-related differences are consistent with previous reports that SHRs display heightened stress reactivity and altered emotional regulation compared with normotensive controls (Hård E et al., 1985). In particular, the pronounced responses to blue and white light may be linked to the spectral sensitivity of intrinsically photosensitive retinal ganglion cells (ipRGCs), which strongly drive non-visual effects of light including circadian regulation, autonomic modulation, and anxiety-related behavior (Berson et al., 2002, LeGates et al., 2014). These findings suggest that a genetic predisposition to hypertension may amplify light-induced behavioral and cardiovascular responses, highlighting the importance of considering both strain background and spectral properties in translational light research.

Rats possess two distinct types of cone photopigments that contribute to their color perception: UV light–sensitive cones and middle-wavelength cones. UV light–sensitive cones exhibit peak sensitivity at approximately 358–359 nm, enabling rats to perceive ultraviolet light, which is beyond the visible spectrum for humans. Middle-wavelength cones are maximally responsive to wavelengths of approximately 509–510 nm, corresponding to the green region of the visible spectrum (Jacobs et al., 2001). Behavioral studies have demonstrated that rats can differentiate between these wavelengths, indicating a rudimentary form of dichromatic color vision. Thus, we selected red, green, and blue wavelengths ranging from 450 to 570 nm and 620–750 nm, which fall within the detectable spectrum for rats and correspond to the primary colors in human vision. Under low-illumination conditions, SHRs exhibited significantly altered activity levels, compared with the observations under the dark condition, only under blue light. By contrast, under high-illumination conditions, no significant differences were observed under red light. These results align with those of Jacobs, who indicated that rats exhibit the lowest sensitivity to red light (620–750 nm) (Jacobs et al., 2001).

We found that SHRs entered the central square of the open field more frequently than did WKY rats across all light conditions; this finding suggests a lower level of anxiety in SHRs than in WKY rats (Nam et al., 2014). However, this observation contrasts with evidence indicating that individuals with ADHD often experience comorbid anxiety (Elahi et al., 2025). A possible explanation for this discrepancy is that although anxiety and ADHD frequently co-occur, not all patients with ADHD exhibit anxiety symptoms. Additionally, the inherent limitations of animal models in fully replicating all ADHD symptoms might have contributed to this discrepancy. Our findings suggest that although SHRs are commonly used to model the hyperactive component of ADHD, they may fail to capture the anxiety component. Consequently, these results imply that SHRs are more representative of a subtype of ADHD that lacks comorbid anxiety.

In the open field test, SHRs exhibited a significant reduction in the number of entries into the central square when exposed to blue light—the shortest wavelength detectable by humans and known for its potential adverse effects on human vision (Zhang et al., 2023). This reduction in exploratory behavior suggests an increase in anxiety-like behaviors in SHRs under blue light. Additionally, heart rate, a key physiological marker of anxiety, was significantly elevated in SHRs under blue light. By contrast, no such effect was observed in WKY rats. These findings collectively indicate that SHRs exhibit higher sensitivity to the anxiogenic effects of blue light than do WKY rats.

Clinical studies have indicated that individuals with ADHD often exhibit heightened sensitivity to light or photophobia, a phenomenon potentially associated with the function of intrinsically photosensitive retinal ganglion cells (ipRGCs). These cells contain the photopigment melanopsin (DeCarlo et al., 2016, Gao et al., 2022, Kooij and Bijlenga, 2014) and play a crucial role in regulating circadian rhythms and modulating the pupillary light reflex, with a peak sensitivity to blue light wavelengths (Reiter and Richardson, 1992, Sanches et al., 2023). While melanopsin exhibits peak sensitivity at approximately 480 nm, its action spectrum is broad and extends from ∼420–500 nm (Berson et al., 2002, Hattar et al., 2002). Notably, ipRGCs project to various brain regions involved in anxiety and stress responses, including the suprachiasmatic nucleus and limbic structures. Although direct evidence of strain-specific differences in ipRGC function is lacking, the increased sensitivity of SHRs to blue light may exacerbate their anxiety-like behaviors. In addition to ipRGC-mediated pathways, cellular-level alterations in the retina may further explain strain differences: SHRs have been reported to undergo progressive photoreceptor loss, particularly affecting rods, due to chronic hypertension–induced oxidative stress and microvascular impairment, whereas WKY rats preserve normal retinal architecture (Lee M et al., 2024). Such structural differences could amplify the functional impact of light exposure on SHRs. Collectively, the findings indicate that blue light exposure significantly reduces exploratory behavior in SHRs, indicating an increase in anxiety, potentially mediated by the modulation of neural circuits involved in emotional processing and stress regulation. This finding is consistent with those of studies demonstrating that SHRs exhibit hyperarousal and heightened activation of the hypothalamic–pituitary–adrenocortical axis in response to stress (Roman et al., 2004, Schaefer et al., 1978). These characteristics may contribute to their pronounced susceptibility to environmental light conditions.

One limitation of this study is the use of a fixed order of light presentation (dark → white → blue → green → red), which may introduce potential carryover or habituation effects. The decision to use a fixed sequence was based on the need to standardize the experimental conditions across subjects and minimize confounding variability from random orderings, especially given the physiological sensitivity of the ECG recordings. Nevertheless, it is possible that prior exposure to a specific light condition may have influenced responses to subsequent conditions. For instance, the initial exposure to darkness may have heightened arousal to subsequent brighter stimuli, or repeated exposures may have induced habituation over time. Future studies could address this limitation by employing a counterbalanced or randomized light presentation order to systematically control for potential sequence effects. Despite this limitation, the consistency of behavioral and physiological responses observed across animals suggests that the core effects of light color and intensity remain robust under the current protocol.

In conclusion, this study demonstrates that short-term exposure to different light spectra, particularly blue light, acutely modulates anxiety-like behaviors, locomotor activity, and autonomic function in rats with high baseline activity. These findings highlight the sensitivity of behavioral and physiological responses to light environments, even over brief durations. Given that this was an acute exposure experiment, the results are particularly relevant to transient settings such as public transportation cabins, clinic waiting rooms, or other short-stay environments where lighting conditions may influence emotional or stress-related responses. Designing such spaces with thoughtful consideration of light color and intensity may help reduce physiological arousal or anxiety-like reactions in sensitive individuals. Further studies are warranted to examine how light-driven behavioral modulation unfolds across varying exposure durations and across different populations or contexts.

## Ethical Standards statement

Animal care, housing, and all experimental procedures adhered to the Codes for Experimental Use of Animals outlined by the Council of Agriculture, Taiwan, on the basis of the Animal Protection Law, Taiwan. The study protocol was approved by the Institutional Animal Care and Use Committee of National Ilan University (Approval No. 103–37). All efforts were made to minimize the number of animals used and to reduce their discomfort throughout the study.

## CRediT authorship contribution statement

**Wen-Yi Wu:** Formal analysis, Validation, Visualization, Writing – original draft, Writing – review & editing. **Shih-Han Liao:** Data curation, Formal analysis, Validation, Writing – original draft. **Teng-Ching Yin:** Formal analysis, Methodology, Visualization. **Meng-Li Tsai:** Conceptualization, Investigation, Methodology, Resources, Validation, Writing – review & editing.

## Declaration of Competing Interest

The authors declare that they have no known competing financial interests or personal relationships that could have appeared to influence the work reported in this paper.
