# Supplementary material for: Effects of different light spectra on locomotion, anxiety, and heart rate in spontaneously hypertensive rats and Wistar–Kyoto rats
Source: IBRO Neurosci Rep. 2025 Nov 19;19:988–95. doi: 10.1016/j.ibneur.2025.11.008 (PMC12681973; doi:10.1016/j.ibneur.2025.11.008)
Supplement: Supplementary file 1 — Supplementary material [file mmc1.docx]

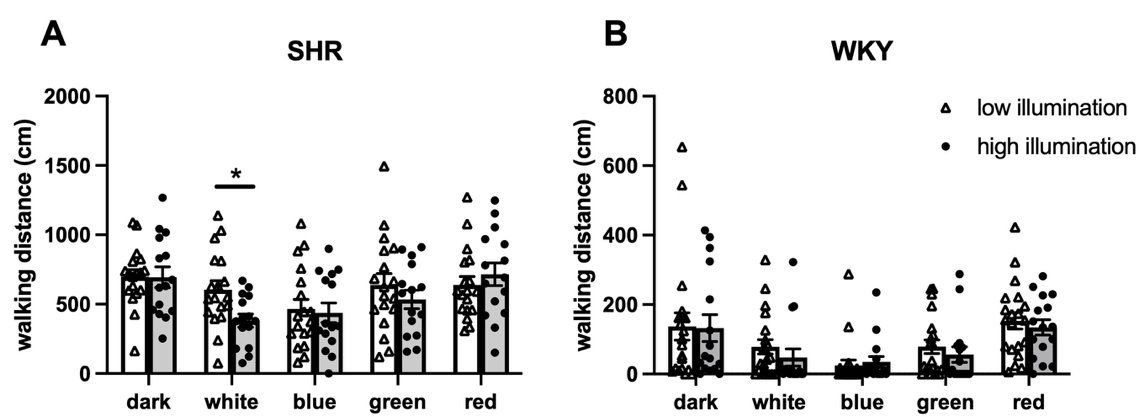


**Figure S1.** Motor activity in SHRs (A) and WKY rats (B) under low (white bar) and high (black bar) illumination across different colored light conditions. (A) In SHRs, the walking distance was significantly lower under white light than under the dark condition under both low (*n* = 18) and high (*n* = 15) illumination. (B) Walking distances in WKY rats under low (*n* = 20) and high (*n* = 16) illumination. Data are presented in terms of mean ± SEM values. **p* < .05, compared with the dark condition.


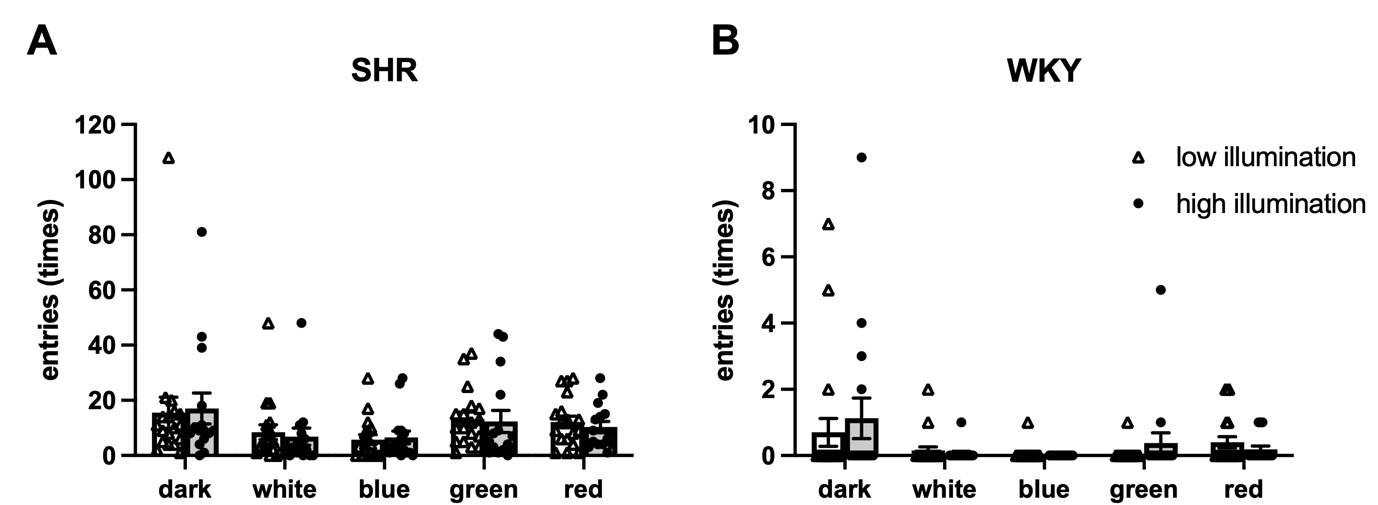


**Figure S2.** Frequency of entries into the central zone of the open field in SHRs (A) and WKY rats (B) under low (white bar) and high (black bar) illumination across different colored light stimuli. (A) In SHRs (*n* = 18 for low illumination, *n* = 15 for high illumination), no significant differences in the frequency of entries into the central zone were observed between low- and high-illumination conditions across any of the colored light conditions. (B) In WKY rats (*n* = 20 for low illumination, *n* = 16 for high illumination) no significant differences were observed across low- and high-illumination conditions, irrespective of the colored light stimulus. Data are presented in terms of mean ± SEM values.


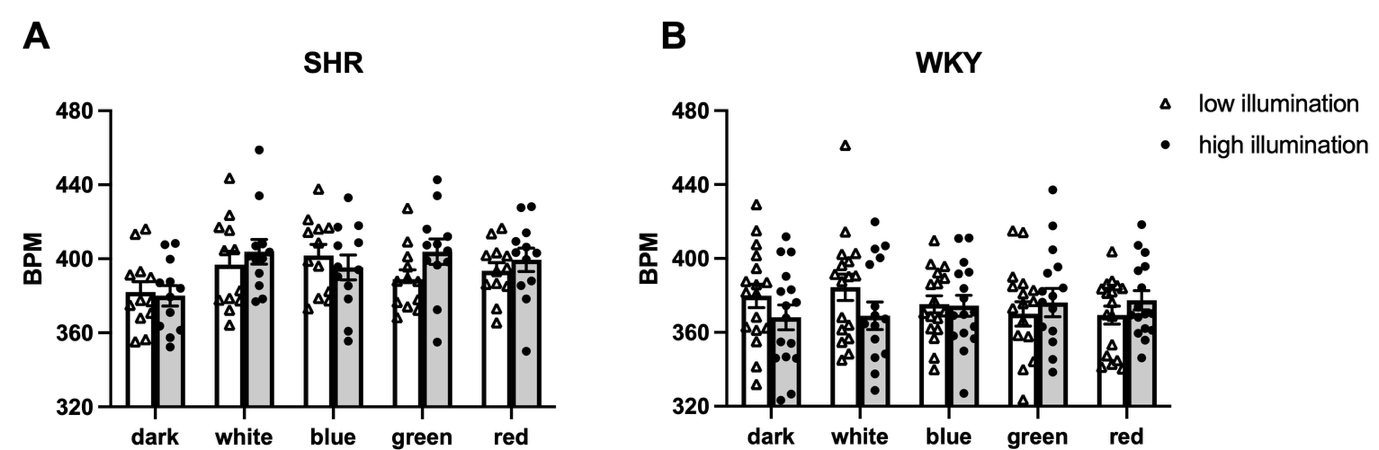


**Figure S3.** Effect of light intensity on heart rate in SHRs (A) and WKY rats (B). (A) In SHRs (*n* = 15 for low illumination; *n* = 12 for high illumination), heart rate did not differ significantly between low- and high illumination across the colored light conditions. (B) In WKY rats (*n* = 17 for low illumination; *n* = 16 for high illumination) no significant differences were observed in heart rate between low- and high illumination across the colored light conditions. Data are presented in terms of mean ± SEM values.
